# Inhibition of growth, biofilm formation, virulence, and surface attachment of *Agrobacterium tumefaciens* by cinnamaldehyde derivatives

**DOI:** 10.3389/fmicb.2022.1001865

**Published:** 2022-10-11

**Authors:** Bilal Ahmed, Afreen Jailani, Jin-Hyung Lee, Jintae Lee

**Affiliations:** School of Chemical Engineering, Yeungnam University, Gyeongsan, South Korea

**Keywords:** *Agrobacterium tumefaciens*, biofilm, cinnamaldehyde, plant, virulence

## Abstract

*Agrobacterium tumefaciens*, a soil-borne, saprophytic plant pathogen that colonizes plant surfaces and induces tumors in a wide range of dicotyledonous plants by transferring and expressing its T-DNA genes. The limited availabilities and efficacies of current treatments necessitate the exploration of new anti-*Agrobacterium* agents. We examined the effects of *trans*-cinnamaldehyde (*t*-CNMA) and its derivatives on the cell surface hydrophobicity, exopolysaccharide and exo-protease production, swimming motility on agar, and biofilm forming ability of *A. tumefaciens*. Based on initial biofilm inhibition results and minimum inhibitory concentration (MIC) data, 4-nitro, 4-chloro, and 4-fluoro CNMAs were further tested. 4-Nitro, 4-chloro, and 4-fluoro CNMA at ≥150 μg/ml significantly inhibited biofilm formation by 94–99%. Similarly, biofilm formation on polystyrene or nylon was substantially reduced by 4-nitro and 4-chloro CNMAs as determined by optical microscopy and scanning electron microscopy (SEM) and 3-D spectrum plots. 4-Nitro and 4-chloro CNMAs induced cell shortening and concentration- and time-dependently reduced cell growth. Virulence factors were significantly and dose-dependently suppressed by 4-nitro and 4-chloro CNMAs (*P* ≤ 0.05). Gene expressional changes were greater after 4-nitro CNMA than *t*-CNMA treatment, as determined by qRT-PCR. Furthermore, some genes essential for biofilm formation, motility, and virulence genes significantly downregulated by 4-nitro CNMA. Seed germination of *Raphanus sativus* was not hindered by 4-nitro or 4-fluoro CNMA at concentrations ≤200 μg/ml, but root surface biofilm formation was severely inhibited. This study is the first to report the anti-*Agrobacterium* biofilm and anti-virulence effects of 4-nitro, 4-chloro, and 4-fluoro CNMAs and *t*-CNMA and indicates that they should be considered starting points for the development of anti-*Agrobacterium* agents.

## Introduction

*Agrobacterium tumefaciens* is a fatal plant pathogenic bacterium responsible for crown-gall disease and contains a Ti-plasmid that is inserted into the plant genome via horizontal gene transfer (Liu and Nester, 2006). This exclusive feature of *A. tumefaciens* has been well researched and utilized for genetic transformations of plants under laboratory conditions (Nguyen et al., 2021). However, in natural environments, pathogenic agrobacteria may infect a range of important crop plants based on their biovars; (i) *A. tumefaciens* species complex (biovar I), (ii) *Agrobacterium rhizogenes* (biovar II), and (iii) *Agrobacterium vitis* (biovar III) (Slater et al., 2009). Of these, *A. tumefaciens* is predominantly found living a saprophytic lifestyle in different environments, including the rhizosphere where it thrives, forms biofilms (Heindl et al., 2014), and may infect a broad range of dicotyledonous plant species (>600), induce gall formation, and cause huge crop losses (>5% of economically important crops globally) (Moens, 2009). *A. tumefaciens* senses chemical signals (rhizospheric signal molecules), such as sugars, organic acids, and amino acids by chemotaxis, and enters host tissues at surface wounds (Liu and Nester, 2006). Furthermore, on receiving signals from plants, *Agrobacterium* increases the expressions of its virulence genes. The two-component VirA/VirG regulatory system activates virulence genes and assists transfer of Ti-plasmid to host plants (Nabi et al., 2022). Virulent *A. tumefaciens* transfers and integrates its T-DNA fragment from a Ti-plasmid into the host genome and its subsequent expression increases the production of opines and plant hormones like cytokinin and indole-3-acetic acid (Dessaux and Faure, 2018; Nabi et al., 2022), which enhance the plant growth and induce tumor formation. Opines are utilized by *Agrobacterium* as nutrients and activate quorum sensing (QS) signaling, which further enhances *A. tumefaciens* virulence and opine metabolism (Faure and Lang, 2014).

For disease to occur, *A. tumefaciens* must first physically attach to the host surface, which may occur in a stepwise manner, as follows: (i) initial surface contact by motile flagella, (ii) establishment of transient reversible attachment facilitated by protein adhesins and a range of pili (conjugative and Ctp) (Matthysse, 2014), and (iii) irreversible attachment by bacterial exopolysaccharides (EPS) after biofilm establishment (Thompson et al., 2018). *A. tumefaciens* can colonize and form biofilms on various abiotic surfaces, plant roots, and wounds, which it reaches by swimming using six flagella located around a single pole (Merritt et al., 2007) and then attaches firmly to cellulose fibrils. Bacterial EPS secretion, pili activity, and biofilm formation vary among soil-borne pathogenic bacteria but are required when pathogens transit to the biofilm mode from planktonic (Muhammad et al., 2020). Generally, antibiotics, copper bactericides, or fosetyl-aluminum are used to control plant pathogenic bacteria, but these measures are less effective at controlling *A. tumefaciens* infections (Lee et al., 2020), not cost-effective, and not readily available. Agrocin 84 (a biopesticide) produced by genetically modified (GM) *Agrobacterium radiobacter* (non-pathogenic) strains K84 and K1026, which competitively colonized the roots of several crops, were reported to inhibit the production of leucyl-tRNA synthetase in *A. tumefaciens* (McCardell and Pootjes, 1976; Kerr and Bullard, 2020). However, its effects on non-targeted useful rhizospheric organisms have not been assessed, and its interactions with agrobacterial species complex (biovar I–III) are unknown. Furthermore, field applications of GM organisms are prohibited in some countries (Kahla et al., 2017). Thus, other novel approaches are needed to prevent *A. tumefaciens* biofilm formation and virulence and control crown gall disease.

Plant-derived bioactive compounds offer a potential source of anti-*Agrobacterium* molecules, and cinnamaldehydes derived from the bark of ∼250 species belonging to the genus *Cinnamomum* are of particular interest (Shreaz et al., 2016). The cinnamaldehyde obtained from essential oils has been categorized as generally regarded as safe (GRAS) by the U.S. Food and Drug Administration (FDA) and has been approved for used in foods (Wei et al., 2011) and given status “A” by the Council of Europe for use in food (Friedman, 2017). Due to its characteristic aroma, color, and taste, *trans*-cinnamaldehyde (*t*-CNMA) is used medically and as a flavoring agent (Chun et al., 2013). Furthermore, *t*-CNMA has been reported to have anti-QS (Zhang et al., 2018), antibiofilm (Yu et al., 2020), and antibacterial effects (Yossa et al., 2014) against several food and clinical pathogens including *Erwinia carotovora* (Zhang et al., 2018), *Pseudomonas fluorescens* (Zhang et al., 2018), *Campylobacter* spp. (Yu et al., 2020), *Escherichia coli* O157:H7 (Yossa et al., 2014), *Salmonella* (Yossa et al., 2014), *Staphylococcus aureus* (Ferro et al., 2016), and the fungus *Candida albicans* (Chen et al., 2019). Moreover, cinnamaldehydes also have antioxidant, anti-inflammatory, anticancer, and anti-diabetic activities (Rao and Gan, 2014). Nevertheless, the antivirulence and antibiofilm effects of *t*-CNMA and its derivatives on *A. tumefaciens* have not been investigated. We hypothesized that *t*-CNMA and its derivatives might inhibit biofilm formation by *A. tumefaciens* on abiotic and biotic surfaces, and thus, we investigated the antibacterial and antibiofilm effects of *t*-CNMA and ten of its derivatives.

## Materials and methods

### Cinnamaldehyde and derivatives

*Trans*-cinnamaldehyde (99%) and 10 of its derivatives: cinnamaldehyde oxime (95%), α-methylcinnamaldehyde (95%), 2-methoxycinnamaldehyde (95%), 2-nitrocinnamaldehyde (98%), 4-bromocinnamaldehyde (95%), 4-methoxycinnamaldehyde (95%), 4-nitrocinnamaldehyde (95%), 4-dimethylaminocinnamaldehyde (98%), 4-fluorocinnamaldehyde (97%), and 4-chlorocinnamaldehyde (95%) were purchased from Sigma-Aldrich (St. Louis, MO, USA) or Combi-Blocks (San Diego, CA, USA). Their molecular weights and chemical structures are given in Table 1. Stocks of 100 mg/ml were prepared in dimethyl sulfoxide (DMSO) and kept at −20°C until required. A total of 0.1% (v/v) DMSO was used as the control for antibacterial and biofilm experiments; at this concentration DMSO did not effect on bacterial growth or biofilm formation.

**TABLE 1:** Minimum inhibitory concentration and biofilm reduction by *t*-CNMA derivatives against *A. tumefaciens*.

Test compound	Structure	MIC (μg/ml)	Biofilm reduction (%) after 48 h	
			100 μg/ml	200 μg/ml
*t*-CNMA		400	16.2	93
	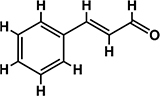			
CNMA oxime		200	24.4	93
	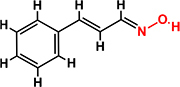			
Alpha-methyl CNMA		>400	4.3	7.4
	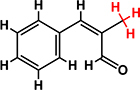			
2-Methoxy CNMA		400	18.4	30.6
	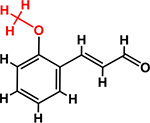			
2-Nitro CNMA		400	26.8	23.5
	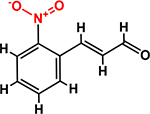			
4-Bromo CNMA		200	10.7	100
	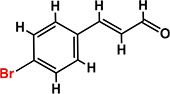			
4-Methoxy CNMA		400	0.0	0.0
	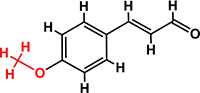			
4-Nitro CNMA		100	91.5	100
	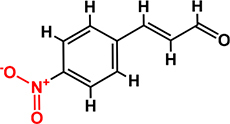			
4-Dimethylamino CNMA		>400	0.0	0.0
	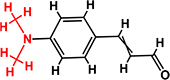			
4-Fluoro CNMA		400	0.0	0.0
	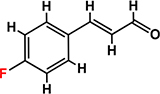			
4-Chloro CNMA		200	4.6	94
	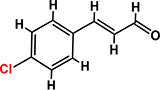			

### Organism and growth conditions

*Agrobacterium tumefaciens* GV2260 was maintained at 30°C on Luria Bertani (LB) agar plates, and for long-term preservation, glycerol (20% v/v) culture stocks in LB were stored at −80°C. For working cultures, two independent colonies from LB agar plates were inoculated in LB broth and incubated at 250 rpm for 24 h at 30°C. At least two independent cultures replicated into three (*n* = 2 × 3 = 6) were used for experiments.

### Antibiofilm screening and minimum inhibitory concentration determinations

For antibiofilm screening, colonies of *A. tumefaciens* grown for 24 h in LB broth were diluted with fresh LB at 1:50, and *t*-CNMA or its derivatives at 100 or 200 μg/ml in LB were added. Bacterial cells incubated with LB only were considered non-treated controls. Aliquots (300 μl) of these cultures were added to wells of 96-well microtiter plates and incubated for 48 h at 30°C. Then *A. tumefaciens* biofilm formation was checked using a crystal violet assay (Ramey and Parsek, 2006), with modifications; wells were washed three times with sterile distilled water kept at room temperature, plates were air-dried, and 300 μl of 0.1% crystal violet was added to each well. After incubation for 20 min at room temperature, wells were rinsed three times with sterile distilled water, and 300 μl of 95% ethanol was added. After shaking microtiter plates for 1 min in a plate reader, biofilm absorbance was recorded at 570 nm. To determine minimum inhibitory concentrations (MICs), *A. tumefaciens* cells were treated with 0–400 μg/ml of *t*-CNMA or its derivatives in LB diluted at 1:100, and then 300 μl aliquots were incubated in a microtiter plate for 24 h at 30°C. Cell growths were determined by measuring optical density at 620 nm.

### Estimation of *Agrobacterium tumefaciens* biofilm production by crystal violet assay: Quantification and microscopy

Cells of strain GV2260 from 24 h cultures were diluted with LB at 1:50 and 25, 50, 75, 100, 150, 200, or 400 μg/ml of *t*-CNMA, 4-nitrocinnamaldehyde (4-nitro CNMA), 4-chlorocinnamaldehyde (4-chloro CNMA), or 4-fluorocinnamaldehyde (4-fluoro CNMA) were added. Crystal violet biofilm assays were performed as described in section “Antibiofilm screening and minimum inhibitory concentration determinations” above. A total of six wells and two independent bacterial cultures were used for each test concentration. The experiment was terminated after incubation for 48 h at 30°C under static conditions.

For microscopic observation, biofilm formation by *A. tumefaciens* was also challenged in 6-well tissue culture plates using *t*-CNMA, 4-nitro, 4-chloro, or 4-fluoro CNMA. Briefly, 3 ml of bacterial suspensions prepared in LB broth (1:50 culture broth ratio) were mixed with *t*-CNMA or its derivatives (50–200 μg/ml) and added to wells. After incubation for 48 h at 30°C under static conditions, the media containing planktonic cells was carefully removed, and biofilms were rinsed carefully three times with PBS in the wells. Biofilms were then stained with crystal violet (0.1%) for 20 min at room temperature, rinsed with distilled water, and visualized using the iRiS Digital Cell imaging system (Logos Biosystems, Annandale, VA, USA). Micrographs were captured, and color mesh plots were created using ImageJ software.

### Effect of *trans*-cinnamaldehyde and derivatives on biofilm formation on membranes

*Agrobacterium tumefaciens* was allowed to form biofilm on nylon membrane surfaces in 96-well plates (Lee et al., 2011). *t*-CNMA, 4-nitro CNMA, 4-chloro CNMA, or 4-fluoro CNMA (200 μg/ml) were added to bacterial cultures in LB (1:50), and small autoclaved pieces of nylon membrane were added to wells. Plates were incubated for 48 h at 30°C. Membranes were then removed, rinsed with sterile PBS, fixed in a mixture of 2.5% glutaraldehyde and 2% formaldehyde solution in distilled water, left for 30 min at room temperature, and then kept at 4°C overnight (Ahmed et al., 2021). Samples were then dehydrated using an ethanol gradient (30, 50, 70, 90, and 100% for 10 min each), critical point dried, coated with Au or Pt, and visualized by FE-SEM (model S-4200, Hitachi, Tokyo, Japan) at 15 kV. The length of biofilm cells attached to nylon surface were determined by measuring the length of at least 50 cells per test concentration using ImageJ software. A scale was set with the “Analyze” tool of ImageJ and then length of each cell (*n* = 50) was determined in micrometers (μm).

### Virulence factor production by *Agrobacterium tumefaciens*

#### Swimming motility

The swimming motility of *A. tumefaciens* GV2260 was assessed using peptone-agar (1% peptone, 0.25% agar, and 0.5% NaCl) containing *t*-CNMA or its derivatives at 25–200 μg/ml. The bacterial inoculum (1 μl) from overnight grown culture was placed at the center of peptone-agar plates and allowed to stand for 72 h at 30°C (Ahmed et al., 2022). Swim diameters were recorded at 48 and 72 h to check cell migration through agar. Data from three replicates were averaged.

#### Measurement of cell surface hydrophobicity

*Agrobacterium tumefaciens* was exposed to *t*-CNMA or its derivatives at 25, 50, 75, 100, 150, 200, or 400 μg/ml in 1 ml LB at a culture: medium ratio of 1:100 in Eppendorf tubes and incubated at 250 rpm for 24 h at 30°C. Microfuge tubes were then centrifuged at 10,000 rpm for 10 min, and cell pellets were mixed with PBS, washed three times, and resuspended in PBS (1 ml). Optical density (OD) values of suspensions were read at 600 nm and designated A_0_. The method described earlier for bacterial adhesion to hydrocarbons (BATH) was followed (Rosenberg, 1984) with modifications. Hexadecane was then added to cell suspensions and vigorously vortexed for 1 min and then left for 30 min at room temperature to allow phase separation. Similarly, a blank (1 ml PBS only) was also processed. Aqueous phase OD (600 nm) values were designated A_i._ Percent hydrophobicity were calculated using the formula:


$$\mathrm{Percent}\mathrm{hydrophobicity}\left(\%H\right)=\frac{A\,0-A\,i}{A\,i}\times 100$$

#### Assessment of extracellular protease production

*Agrobacterium tumefaciens* culture mixed with LB at 1:100 was exposed to *t*-CNMA compounds at 25–400 μg/ml in LB at 250 rpm for 24 h at 30°C. Samples were then spun at 10,000 rpm for 10 min, and supernatants were collected. Supernatants (100 μl) were mixed with an equal volume of azocasein and incubated for 30 min at 37°C when 600 μl of tricarboxylic acid (10%) was added to stop proteolysis. These mixtures were then kept for 30 min at −20°C. After centrifugation at 10,000 rpm for 10 min, 700 μl of the resulting supernatants was added to 700 μl of NaOH, and absorbances were recorded at 440 nm (Sethupathy et al., 2020).

#### Determination of exopolysaccharides production

*Agrobacterium tumefaciens* was grown with or without *t*-CNMA derivatives at 25, 50, 75, 100, 150, 200, or 400 μg/ml in LB in 1.5 ml microfuge tubes at 250 rpm for 24 h at 30°C. Tubes were centrifuged at 10,000 rpm for 10 min. Supernatants were added with chilled ethanol at a ratio of 1:3 and left undisturbed at 4°C for overnight. EPS precipitates were collected by centrifugation (10,000 rpm for 5 min.) and solubilized in 200 μl of water. A phenol/sulfuric acid mixture (prepared at a ratio of 1:5) was then added to 200 μl of EPS samples, incubated for 30 min. at room temperature, and left at room temperature for 20 min. Absorbances were measured at 490 nm (Ali et al., 2016).

### Effect of cinnamaldehyde derivatives on the planktonic cell growth of *Agrobacterium tumefaciens*

#### Time-dependent growth inhibition assay

*Agrobacterium tumefaciens* was grown in LB diluted at 1:100 for 24 h then treated with *t*-CNMA or its derivatives at different concentrations. These suspensions (300 μl) were then added to the wells of a 96-well plate. Culture growths were monitored every 2 h at 620 nm for 24 h (Ahmed et al., 2019b). The results of two independent cultures in six wells per concentration were averaged and plotted as a function of incubation time and concentration.

#### Impact of *trans*-cinnamaldehyde derivatives on CFU count and percent cell survival

*Agrobacterium tumefaciens* grown for 24 h in LB was diluted at 1:100 with 25–400 μg/ml of *t*-CNMA, 4-nitro CNMA, 4-chloro CNMA, or 4-fluoro CNMA and incubated at 250 rpm for 24 h at 30°C. Aliquots (100 μl) of appropriate dilutions were then plated on LB agar and incubated for 48 h at 30°C. Colonies were counted, and CFU/ml values were calculated and converted to a log scale:


$$C\,F\,U/m\,l=\frac{N\,o.o\,f\,c\,o\,l\,o\,n\,i\,e\,s\,c\,o\,u\,n\,t\,e\,d\times D\,i\,l\,u\,t\,i\,o\,n\,f\,a\,c\,t\,o\,r}{V\,o\,l\,u\,m\,e\,p\,l\,a\,t\,e\,d\,\left(m\,l\right)}$$

Survival percentages of *A. tumefaciens* were also calculated with respect to non-treated controls.

### Biofilm formation by *trans*-cinnamaldehyde derivatives-challenged *Agrobacterium tumefaciens* on root surfaces

Seeds of *Raphanus sativus* were germinated on 0.86 g/L MS medium supplemented with 0.7% agar, and after germination, seedlings were grown for 5 days. Seedling roots (*n* = 5 per test concentration) were placed in 6-well plates in an aseptic environment. Inoculums of *A. tumefaciens* prepared in 4 ml LB broth at a 1:50 ratio and treated with 200 μg/ml of *t*-CNMA, nitro CNMA, 4-chloro CNMA, or 4-fluoro CNMA. Biofilm development was initiated at 30°C, and experiments were terminated after 48 h of incubation. Growth media containing planktonic cells and loosely attached biofilms were removed by rinsing the roots with sterile PBS. Root samples were then fixed in 2.5% glutaraldehyde and 2% paraformaldehyde for 30 min at room temperature and then overnight at 4°C. Fixatives were removed, and samples were rinsed with PBS, dehydrated using a graded ethanol series (30, 50, 70, 90, and 100%) for 10 min, and kept in isoamyl acetate. Root samples were dried in a critical point dryer (CPD), sputter-coated with gold or platinum, subjected to SEM (S-4200 Hitachi FE-SEM) at 15 kV, and photographed at different magnifications.

### qRT-PCR analysis of *Agrobacterium tumefaciens* genes

To assess the expressional changes induced by *t*-CNMA and 4-nitro CNMA, qRT-PCR was used to analyze the expressions of motility (*flgE* and *motA*), biofilm (*celA, cheA*, and *phoB*), virulence (*virE2, chvE, virE0*, and *virG*), stress related response (*clpB, dnaK, gsp, marR, soxR*, and *hspAT2*), and efflux pump (*emrA, norM, ifeA*, and *ifeR*) genes. *A. tumefaciens* culture in 25 ml LB with an OD_600_ *_nm_* of 1.0 was incubated with 100 μg/ml of *t*-CNMA or 4-nitro CNMA at 250 rpm for 8 h at 30°C. An RNase inhibitor (700 μl; RNAlater, Ambion, TX, USA) was added and gently agitated on ice. Centrifugation of untreated and treated cultures was performed at 13,000 rpm for 10 min at 4°C. The RNA was extracted using the Qiagen RNeasy mini kit (Valencia, CA, USA) and concentrations were determined using a nanodrop spectrophotometer (model: Cytiva NanoVue Plus Spectrophotometer, Fisher Scientific, England, UK). Primer sequences of tested genes are provided in Supplementary Table 1. qRT-PCR was conducted using SYBR green master mix and an ABI StepOne Real-Time PCR System (Applied Biosystems, Foster City, CA, USA) using two independent cultures (Lee et al., 2015).

### Evaluation of seed germination

The impact of *t*-CNMA and its derivatives on white radish (*R. sativus*) seed germination was examined (Ahmed et al., 2019a). In brief, *R. sativus* seeds were soaked in distilled water for 6 h, thoroughly rinsed with distilled water and then ethanol (95%), and surface sterilized using sodium hypochlorite (3% for 10 min). *t*-CNMA its derivatives at 25–400 μg/ml were added to soft agar (0.7% agar) containing 0.86 g/L Murashige and Skoog (MS) medium. After washing with autoclaved distilled water, 10 seeds/plate/test were placed and incubated at 25°C for 4 days. The seeds showing evidence of germination were counted.

### Data analyses

All experiments were performed with two independent bacterial cultures. Data are expressed as means ± standard deviations (SD), and significances of differences were determined using the two-tailed *t*-test. Statistical significance was accepted for *P*-values ≤ 0.05 unless otherwise stated. Graphs were prepared using Sigma Plot Ver. 14.0.

## Results

### Determination of minimum inhibitory concentrations of cinnamaldehydes against *Agrobacterium tumefaciens*

The effects of *t*-CNMA and 10 of its derivatives on *A. tumefaciens* were investigated. As shown in Table 1, MICs were variable. We selected three derivatives, viz. 4-nitro CNMA, 4-fluoro CNMA, and 4-chloro CNMA based on their MICs, which were 100, 200, and 200 μg/ml, respectively, and their biofilm inhibitory effects at 100 or 200 μg/ml (Table 1). *t*-CNMA (MIC 400 μg/ml) was used as the control.

### Biofilm formation of *Agrobacterium tumefaciens* in the presence of *trans*-cinnamaldehydes

**FIGURE 1:**
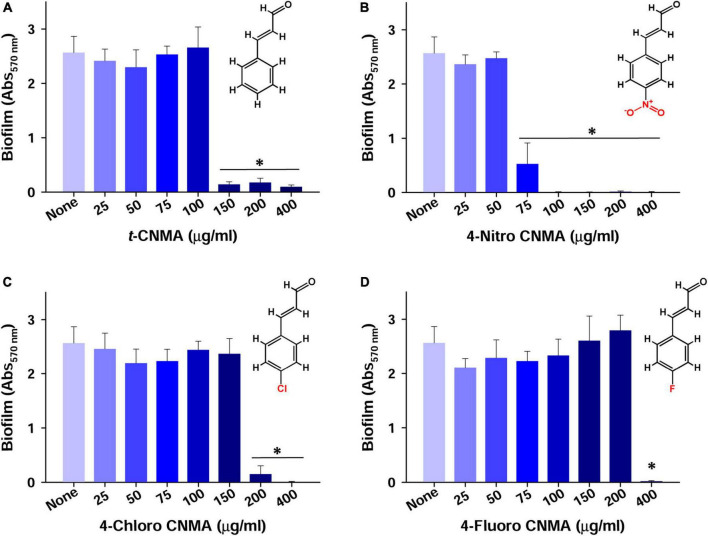
Inhibition of *A. tumefaciens* biofilm formation by *t*-CNMA **(A)**, 4-nitro CNMA **(B)**, 4-chloro CNMA **(C)**, and 4-fluoro CNMA **(D)** after 48 h in polystyrene microtiter plates containing LB. “*” denotes a significant difference by the two-tailed *t*-test between non-treated and treated cultures.

Biofilm formation of *A. tumefaciens* in 96-well plates (Figure 1) was inhibited most by 4-chloro CNMA and 4-nitro CNMA by 94 and 100%, respectively, at 200 μg/ml (Table 1). 4-Fluoro CNMA did not inhibit biofilm at 200 μg/ml but reduced biofilm formation by >90% at 400 μg/ml (Figure 1D) versus the non-treated control. *t*-CNMA reduced biofilm at ≥150 μg/ml, whereas 4-nitro CNMA reduced it significantly (*P* ≤ 0.05) at ≥75 μg/ml (Figure 1B). 4-Chloro CNMA inhibited biofilm formation at 200 μg/ml (*P* ≤ 0.05) (Figure 1C).

### Microscopic assessments of *Agrobacterium tumefaciens* biofilms on polystyrene and nylon surfaces

**FIGURE 2:**
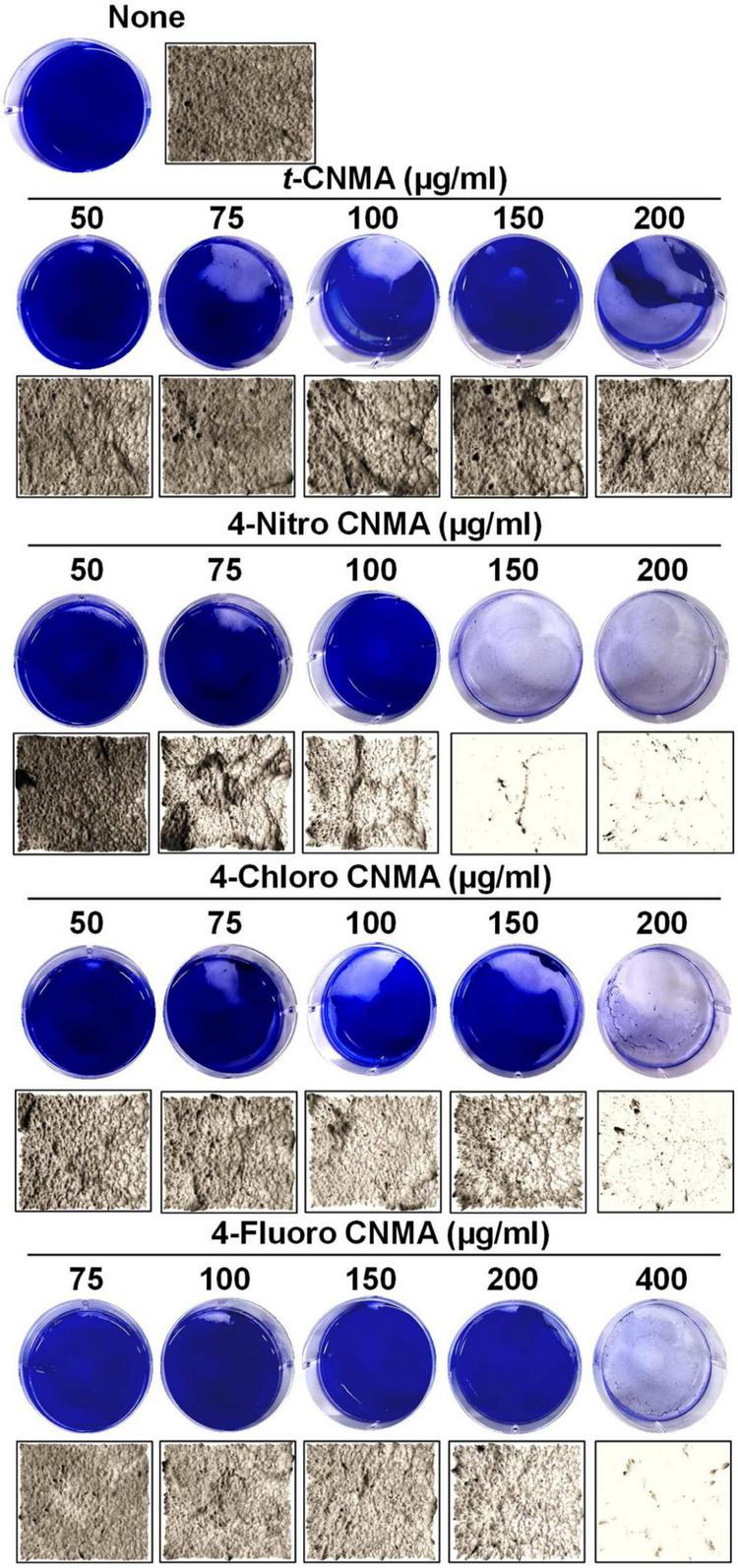
Inhibition of *A. tumefaciens* biofilm formation on flat polystyrene in 6-well plates as determined by crystal violet staining **(A)** and optical microscopy at 20× magnification **(B)** by *t*-CNMA, 4-nitro CNMA, 4-chloro CNMA, and 4-fluoro CNMA in the concentration range (50–400 μg/ml).

**FIGURE 3:**
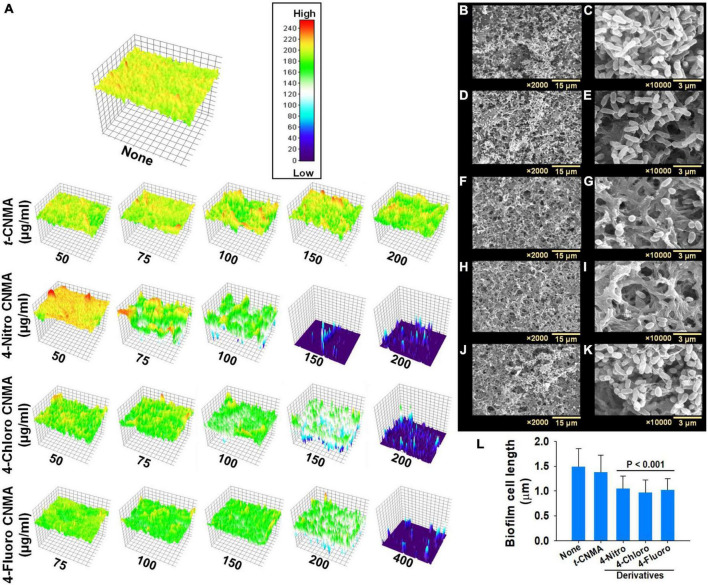
Visual assessment of *A. tumefaciens* biofilm thicknesses formed on polystyrene after exposure to increasing concentrations of *t*-CNMA, 4-nitro CNMA, 4-chloro CNMA, or 4-fluoro CNMA. The colored 3-D images show biofilm thicknesses **(A)** on a low to high (0–200) scale, where red indicates maximum biofilm formation and dark blue no biofilm formation. Biofilms of non-treated **(B,C)** *A. tumefaciens* on nylon and its inhibition by *t*-CNMA **(D,E)**, 4-nitro CNMA **(F,G)**, 4-chloro CNMA **(H,I)**, and 4-fluoro CNMA **(J,K)** after incubation for 48 h. Micrographs taken at 2,000× and 10,000× revealed complete inhibition by 4-nitro and 4-chloro CNMA and reductions in cell lengths **(L)**, although *t*-CNMA **(E)** and 4-fluoro CNMA **(K)** showed biofilm with the presence of EPS threads.

Biofilm formation by *A. tumefaciens* was also investigated on flat polystyrene and nylon membranes. Almost all tested compounds dose-dependently reduced biofilm volumes (Figure 2A), and microscopic images showed they reduced biofilm thicknesses (Figure 2B). *t*-CNMA had the least inhibitory effect at 200 μg/ml, whereas only a few traces of biofilms remained after treatment with 4-nitro CNMA at 150 or 200 μg/ml. 4-Chloro CNMA slightly reduced biofilm formation at 150 μg/ml and completely inhibited it at 200 μg/ml. However, 4-fluoro CNMA did not affect biofilm formation at 200 μg/ml but drastically reduced it at 400 μg/ml. Similarly, 3-D mesh-filled spectrum-LUT plots of biofilms were observed at a scale range of 0–240, after exposing biofilms to different concentrations of *t*-CNMA, 4-nitro CNMA, 4-chloro CNMA, and 4-fluoro CNMA showed dramatic shifts in color to blue at 150–200 μg/ml (Figure 3A). 4-Chloro CNMA (150 μg/ml) and 4-fluoro CNMA (200 μg/ml) had lesser effects on biofilms.

**FIGURE 4:**
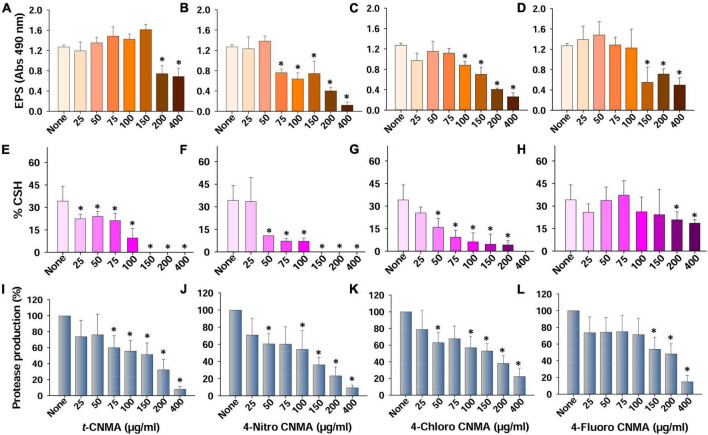
Production of virulence factors and their inhibition by *t*-CNMA and its 4-nitro, 4-chloro, and 4-fluoro CNMA derivatives. Effects on EPS production **(A–D)**, cell surface hydrophobicity **(E–H)**, and extracellular protease production **(I–L)**. “*” denotes a significant difference by the two-tailed *t*-test (*P* ≤ 0.05).

Scanning electron microscopy (SEM) of biofilms on nylon membranes showed variable reductions in biofilm formation by *t*-CNMA (Figures 4D,E), 4-nitro CNMA (Figures 4F,G), 4-chloro CNMA (Figures 4H,I), and 4-fluoro CNMA (Figures 4J,K) versus non-treated controls (Figures 4B,C). The specimens treated with 4-nitro CNMA or 4-chloro CNMA were most affected, and fewer cells were attached to nylon surfaces (Figures 4F–I). In addition, shortening of *A. tumefaciens* cells treated with 4-nitro, 4-chloro, and 4-fluoro derivatives was observed at ×10,000 (Figure 4K). Specifically, the sizes of non-treated *A. tumefaciens* biofilm cells were 1.5 ± 0.4 μm which decreased slightly by 7.5% (1.4 ± 0.3 μm) after *t*-CNMA treatment. However, 4-nitro, 4-chloro, and 4-fluoro significantly (*P* < 0.001) decreased the length by 30% (1 ± 0.25 μm), 35% (0.96 ± 0.25 μm), and 31.3% (1 ± 0.22 μm), respectively (Figure 4L). SEM observations indicated *t*-CNMA and its derivatives alter the morphology and architecture of *A. tumefaciens* biofilms.

### Impact of *trans*-cinnamaldehyde derivatives on exopolysaccharides production and cell surface hydrophobicity

The QS regulates virulence factors considered responsible for *A. tumefaciens* biofilm formation (Faure and Lang, 2014). Of these, cell surface hydrophobicity and extracellular polymeric substances are critical for bacterial adhesion and successful biofilm formation. Non-treated cells of *A. tumefaciens* produced significant amounts of EPS; however, treatments with *t*-CNMA or its derivatives decreased EPS production at relatively high-test concentrations (Figures 4A–D). *t*-CNMA did not reduce the EPS secretion below 150 μg/ml but decreased it significantly by >40% at concentrations ≥200 μg/ml (Figure 4A). 4-Nitro CNMA at 75 μg/ml caused a similar reduction (40%) and at 200 μg/ml decreased EPS production by 69% (Figure 4B). 4-Chloro CNMA caused a significant reduction at 100 μg/ml followed by a concentration-dependent decrease till 400 μg/ml (Figure 4C). The reduction in EPS production by 4-fluoro CNMA (Figure 4D) was similar to that of *t*-CNMA. Cell surface hydrophobicity (% CSH) is essential for bacterial attachment to surfaces (Tribedi and Sil, 2014) and was also found to be decreased by *t*-CNMA and its three derivatives in a concentration-dependent manner (Figures 4E–H). When we compared the inhibitory effects on % CSH at a concentration of 100 μg/ml, 4-nitro CNMA (Figure 4F) and 4-chloro CNMA (Figure 4G) had the greatest effects and reduced CSH by 79% (*P* ≤ 0.05) and 82% (*P* ≤ 0.05), respectively, versus non-treated controls. At 150–400 μg/ml, %CSH was zero for *t*-CNMA (Figure 4E) and 4-nitro CNMA (Figure 4F). As was observed in the EPS assay, 4-fluoro CNMA had least reduction (45% at 200 μg/ml) (Figure 4H).

### Inhibition of protease production and motility by *trans*-cinnamaldehyde derivatives

**FIGURE 5:**
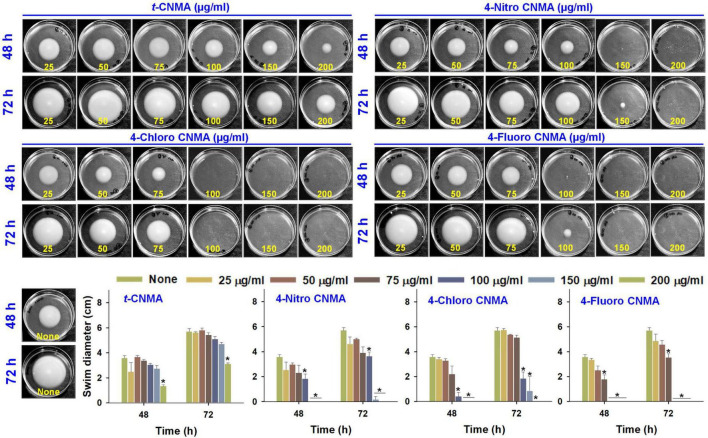
Swimming motility of *A. tumefaciens* on motility agar (1% peptone, 0.25% agarose, and 0.5% NaCl) and its inhibition by *t*-CNMA, 4-nitro CNMA, 4-chloro CNMA, and 4-fluoro CNMA (25–200 μg/ml) after exposure for 48 and 72 h. “*” denotes a significant difference in swim diameter by the two-tailed *t*-test (*P* ≤ 0.05).

Production of exo-proteases by *A. tumefaciens* was dose-dependently reduced by *t*-CNMA and its derivatives (Figures 4I–L). At 100 μg/ml, *t*-CNMA, 4-nitro CNMA, and 4-chloro CNMA significantly reduced the protease production. 4-Nitro CNMA most effectively inhibited protease production. In regards of motility, *A. tumefaciens* exhibited swimming motility on agar plates prepared with 1% peptone, 0.25% agar, and 0.5% NaCl, and this increased with time (48–72 h). The bacterium reached a swimming diameter of 5.7 cm after 72 h incubation in non-treated agar (Figure 5). The addition of *t*-CNMA or its derivatives to agar at 25–200 μg/ml decreased swimming diameters, and no mobility was observed after treatment with 4-nitro CNMA or 4-chloro CNMA at 150 or 100 μg/ml, respectively (Figure 5). Interestingly, 4-fluoro CNMA inhibited swimming motility at 100 μg/ml (*P* ≤ 0.05). Furthermore, *t*-CNMA supplemented agar reduced promoted mobility versus non-treated agar, although even at 200 μg/ml, *A. tumefaciens* exhibited limited mobility (3.1 cm diameter).

### *Trans*-cinnamaldehyde derivatives reduced the planktonic cell growth of *Agrobacterium tumefaciens*

**FIGURE 6:**
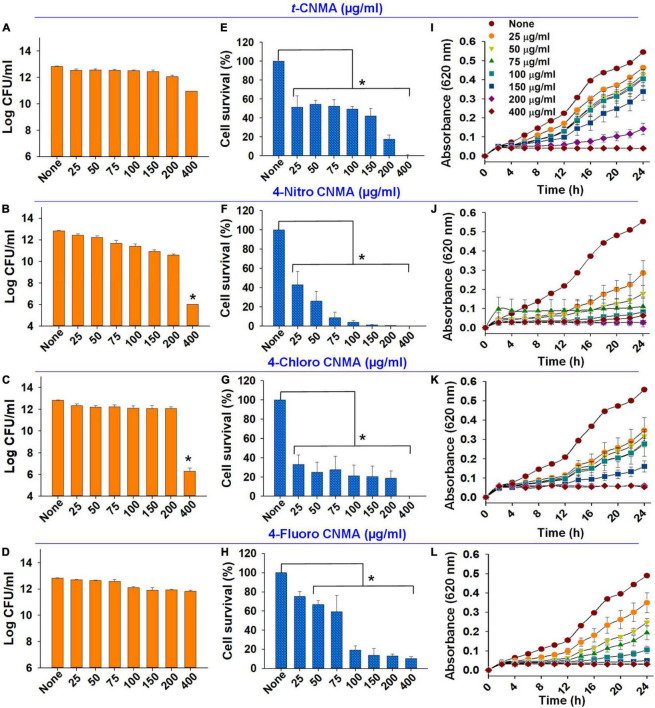
Effects of cinnamaldehydes on *A. tumefaciens* planktonic growth. Log reduction in CFU counts by *t*-CNMA **(A)**, 4-nitro CNMA **(B)**, 4-chloro CNMA **(C)**, and 4-fluoro CNMA **(D)**; percent cell survival after treatment with *t*-CNMA **(E)**, 4-nitro CNMA **(F)**, 4-chloro CNMA **(G)**, or 4-fluoro CNMA **(H)** for 24 h. Time and concentration-dependent growth inhibition were recorded every 2 h **(I–L)**. “*” denotes a significant difference in planktonic growth by the two-tailed *t*-test (*P* ≤ 0.05).

The planktonic cell growth of *A. tumefaciens* was assessed using logarithmic values of CFUs (Figures 6A–D), percent cell survival (Figures 6E–H), and time-dependent growth curves (Figures 6I–L). Total log CFU/ml counts of *A. tumefaciens* grown in the presence of *t*-CNMA (Figure 6A), 4-nitro CNMA (Figure 6B), 4-chloro CNMA (Figure 6C), and 4-fluoro CNMA (Figure 6D) were reduced by only 4-nitro CNMA (>6-log reduction) and 4-chloro CNMA (>6.5-log reduction) at 400 μg/ml. 4-Fluoro CNMA also reduced cell survival, but less than the other derivatives; a fraction of cells survived even after treatment with 4-fluoro CNMA at 400 μg/ml. Concentration (25–400 μg/ml) and time (0–24 h) dependent analysis of *A. tumefaciens* planktonic growth showed *t*-CNMA (Figure 6I), 4-nitro CNMA (Figure 6J), 4-chloro CNMA (Figure 6K), and 4-fluoro CNMA (Figure 6L) induced concentration-dependent decreases. 4-Nitro CNMA and 4-chloro CNMA inhibited cell growth at 150 and 200 μg/ml, respectively (Figures 6J,K). The possible reason for this cell killing could be the enhanced direct contact of cells with tested compound at 250 rpm shaking.

### Reduced biofilm formation on *Raphanus sativus* roots

**FIGURE 7:**
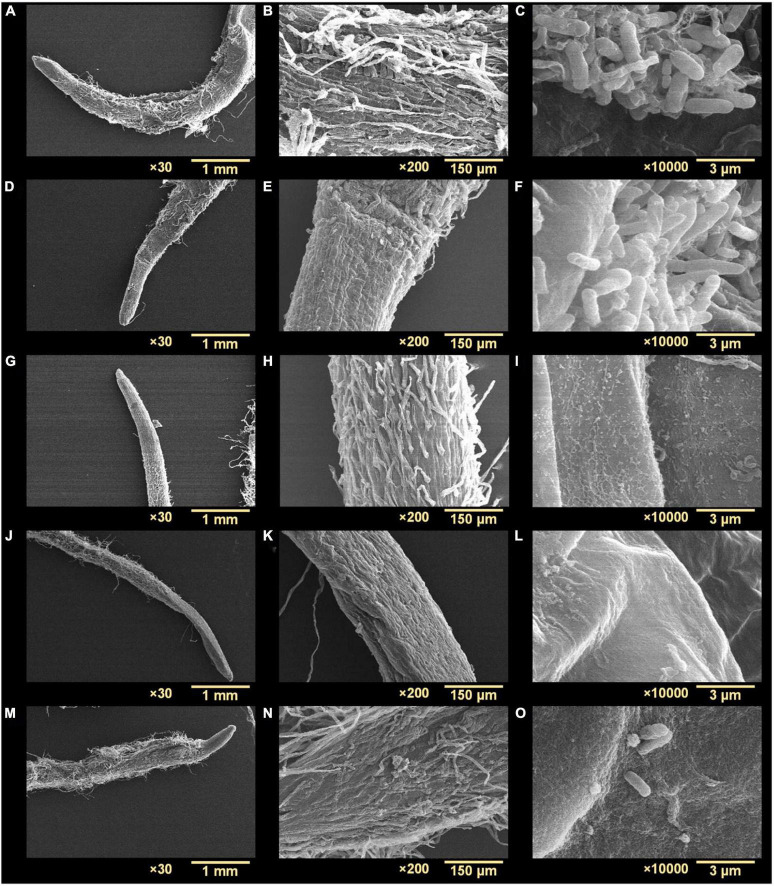
Scanning electron microscopy micrographs of *A. tumefaciens* biofilms on the surface of *R. sativus* at three different magnifications (30×, 200×, and 10,000×): non-treated **(A–C)**, treated with *t*-CNMA **(D–F)**, 4-nitro CNMA **(G–I)**, 4-chloro CNMA **(J–L)**, and 4-fluoro CNMA **(M–O)**.

*Agrobacterium tumefaciens* formed biofilms on *R. sativus* roots after incubation for 48 h under non-treated and optimized growth conditions (Figures 7A–C). The biofilms produced were dense and showed multiple aggregates of cells embedded in an EPS-like substance (Figure 7C). While observing root surfaces, biofilms were observed in multiple regions after *t*-CNMA treatment (Figures 7D–F). However, no biofilms were observed on roots after exposure to 4-nitro CNMA (Figures 7G–I), 4-chloro CNMA (Figures 7J–L), or 4-fluoro CNMA (Figures 7M–O), though a few cells were dispersed at some locations on root surfaces (Figures 7I,O). Furthermore, root surfaces in different zones, e.g., root tips, hairs, and meristematic and root elongation zones were undamaged after exposure to 200 μg/ml of the test compounds. SEM findings after 4-fluoro CNMA treatment (Figures 7M–O) differed from nylon membrane results (Figures 3J,K) since 4-fluoro CNMA did not eradicate biofilms on nylon membranes. On the other hand, results for *t*-CNMA, 4-nitro CNMA, and 4-chloro CNMA treatments of biofilms on nylon (Figure 3) and *R. sativus* root surfaces (Figure 7) were correlated.

### Changes in gene expressions induced by *trans*-cinnamaldehyde and 4-nitro cinnamaldehyde

**FIGURE 8:**
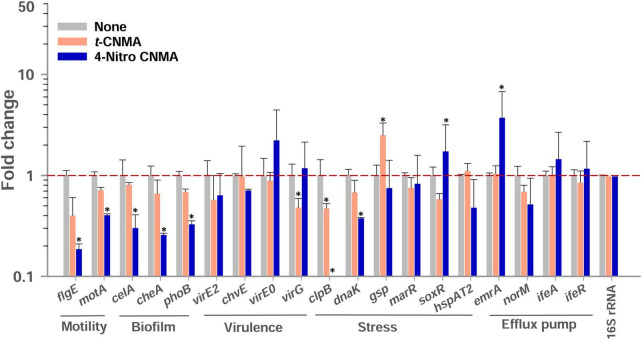
Gene expressional changes in *A. tumefaciens* induced by 100 μg/ml *t*-CNMA or 4-nitro CNMA after treatment in LB broth at 250 rpm for 8 h at 30°C. Bars are averages of four reactions performed using two independent cultures. 16S rRNA was used as the housekeeping gene. “*” represents a significant difference (*P* ≤ 0.05) between non-treated and treated cells. Fold changes in gene expression were calculated using the 2^–ΔΔCt^ method.

Biofilm, virulence, stress, motility, and efflux pump regulation genes of *A. tumefaciens* (OD_600_ *_nm_* = 1.0) assessed after 8 h contact with test compounds in LB at 30°C were variably affected by *t*-CNMA and 4-nitro CNMA versus non-treated controls while 4-nitro CNMA more significantly affected the gene expression than *t*-CNMA (Figure 8). Genes subjected to qRT-PCR were selected based on their direct or indirect involvements with these functions. For example, the biofilm formation genes *cheA*, *celA*, and *phoB* encode for the two-component sensor kinase of the Che operon that regulates chemotaxis (Merritt et al., 2007), cellulose synthase required for cellulose production (Matthysse et al., 2005), and production of a regulatory protein for the two-component (PhoR-PhoB) system (Tomlinson et al., 2010), respectively. 4-Nitro CNMA significantly (*P* ≤ 0.05) downregulated *celA, cheA*, and *phoB* by 3.3-, 3.9-, and 3-fold, respectively, versus non-treated controls (Figure 8). Similarly, two flagellar motility genes *flgE* and *motA*, which encode for a flagellar hook protein and a constituent of the flagellar motor of *A. tumefaciens*, were downregulated by 11- and 2.5-fold, respectively, by 4-nitro CNMA (Merritt et al., 2007). Interestingly, *t*-CNMA only slightly reduced the expression of these genes (Figure 8). Among the virulence genes, *virE2* encodes for virulence protein (virE2) that facilitates the import of T-DNA-protein complex in the host nucleus (Li et al., 2020). *chvE* encodes for a periplasmic-binding protein, which after interacting with the VirA/VirG regulatory system induces the expressions of *vir* genes (Hu X. et al., 2013). *virE0* encodes for a regulator protein that may be directly involved in *Agrobacterium*-plant interactions (Yuan et al., 2008). *virG* encodes for a two-component response regulator protein (Yuan et al., 2008) and was significantly downregulated (Figure 8) by *t*-CNMA only. The expressions of all other virulence genes were either unchanged or non-significantly downregulated. Genes involved in multiple stress responses, namely, *clpB*, *dnaK*, *gsp*, *marR*, and *hspAT2*, reported in other studies (Rosen et al., 2001; Tsai et al., 2012; Rittiroongrad et al., 2016) were also tested. Results revealed that *dnaK* and *clpB* were downregulated by 2.7- and 28-fold, respectively, and *soxR* was upregulated (1.72) by 4-nitro CNMA (the expressions of other stress-related genes were slightly changed or unaffected). *t*-CNMA upregulated the *gsp* gene, which is associated with general stress, by 2.5-fold, and 4-nitro CNMA reduced the expression of a heat shock protein (*hspAT2*). Also, some efflux pump genes, namely, *emrA*, *norM*, *ifeA*, and *ifeR*, were included (Palumbo et al., 1998; Nuonming et al., 2018; Khemthong et al., 2019), but the only effect observed was that 4-nitro CNMA upregulated *emrA* by 3.7-fold (Figure 8).

### *Raphanus sativus* seed germination in the presence of cinnamaldehyde derivatives

The effects of *t*-CNMA, 4-nitro CNMA, 4-chloro CNMA, and 4-fluoro CNMA were also investigated on *R. sativus* seed germination. The impacts of tested compounds at 25–400 μg/ml on percent seed germination were variable (Supplementary Figure 1). Seed germination was non-significantly reduced by *t*-CNMA (Supplementary Figure 1A), 4-nitro CNMA (Supplementary Figure 1B), and 4-fluoro CNMA (Supplementary Figure 1D) at concentrations up to 200 μg/ml. However, 4-chloro CNMA caused 70% inhibition at 200 μg/ml (Supplementary Figure 1C). At 400 μg/ml *t*-CNMA, 4-chloro CNMA, and 4-fluoro CNMA prevented germination, but interestingly, 4-nitro CNMA at this concentration only reduced germination by 41%.

## Discussion

We report the biofilm inhibiting characteristics of *t*-CNMA and ten derivatives, which were selected because of their dissimilar functional moieties on the aromatic ring or side chain of *t*-CNMA. A few studies on CNMA derivatives have reported the antityrosinase effects of α-substituted derivatives such as α-methylcinnamaldehyde, α-chlorocinnamaldehyde, and α-bromocinnamaldehyde (Cui et al., 2015). Similarly, fungal growth was inhibited by 2-bromo and 2-chlorocinnamaldehyde (Badawy and Rabea, 2013). Among the ten derivatives investigated in the present study, 4-nitro CNMA, 4-chloro CNMA, and 4-fluoro CNMA were subjected to further testing because they exhibited potent antibiofilm effects and low MICs. Badawy and Rabea (2013) reported that chitosan-based derivatives had antifungal activity against seven fungal species. The same authors investigated the effects of chitosan-based derivatives, such as N-(α-methylcinnamyl) chitosan and N-(o-methoxycinnamyl) chitosan, on *A. tumefaciens* but reported very high MICs of 1,275 and 1,925 μg/ml, respectively. In the present study, the MICs of 4-nitro CNMA, 4-fluoro CNMA, and 4-chloro CNMA were 100, 200, and 200 μg/ml, respectively. Zhu et al. (2009) previously reported *t*-CNMA thiosemicarbazone had an *A. tumefaciens* MIC of 100 μg/ml with no report on antibiofilm potential and gene expressional changes.

Biofilms provide microorganisms on abiotic and biotic surfaces with well-structured protective sheaths impermeable to drugs and antibacterial agents (Sharma et al., 2019). Thus, microorganisms in biofilms are more virulent than planktonic cells, and novel solutions are required to address this challenge (Ying et al., 2019). *A. tumefaciens* adheres to surfaces using its molecular appendages (Thompson et al., 2018) and subsequently forms reversible or irreversible attachments (Heindl et al., 2014) with abiotic (Figures 2, 3) or biotic surfaces (Figure 7). After establishing contact with a surface, *A. tumefaciens* releases EPS to make this contact reversible and initiates microcolony formation (Heindl et al., 2014). In the present study, 4-nitro CNMA and 4-chloro CNMA significantly inhibited biofilm formation by *A. tumefaciens* on polystyrene and nylon (Figures 2, 3F–I) and plant root surfaces (Figures 7G–L), as determined by light microscopy and SEM. Similar reductions in bacterial aggregation and microcolony formation by *P. fluorescens* were observed by light microscopy after cinnamaldehyde exposure. In a previous study, SEM revealed a maximally disrupted biofilm architecture of *P. fluorescens* at 0.1 μl/ml *t*-CNMA (Li et al., 2018). We observed gaps and poor volumes of *A. tumefaciens* biofilms (Figure 3) after treatments with 4-nitro CNMA (150–200 μg/ml), 4-chloro CNMA (200 μg/ml), or 4-fluoro CNMA (400 μg/ml) but not after treatment with *t*-CNMA. Li et al. (2018) reported that *t*-CNMA induced fissures in *P. fluorescens* biofilms, and Kim et al. (2015) observed cinnamon bark oil and *t*-CNMA at 0.01% v/v reduced enterohemorrhagic *E. coli* (EHEC) fimbriae formation, which is required for biofilm maturation, and suggested that reduced EHEC fimbriae production by *t*-CNMA on nylon membranes was largely responsible for biofilm inhibition. Our observations of reductions in *A. tumefaciens* biofilm formation at ≥150 μg/ml by *t*-CNMA, 4-nitro CNMA, and 4-chloro CNMA (Figure 1) suggest that –NO_2_ functional group at the fourth position on the aromatic ring is more detrimental to biofilm formation than –Cl, and that –F is less effective than –NO_2_ or –Cl. When we compared the gene expressional changes induced by 4-nitro CNMA and *t*-CNMA, 4-nitro CNMA was found to have substantially more potent effects (Figure 8). Furthermore, our findings regarding the effects of *t*-CNMA concur with those of Budri et al. (2015) who found that at 106 μg/ml *t*-CNMA reduced MRSA biofilm formation on stainless steel and polystyrene by 45 and 70%, respectively. Similarly, Albano et al. (2019) found *t*-CNMA at 300 μg/ml reduced biofilm formation by *Staphylococcus epidermidis* by 89%.

Biofilm inhibition by *t*-CNMA might be related to disruption of the QS regulatory system, as has been reported for *P. fluorescens* (Li et al., 2018) and *E. coli* (Niu et al., 2006), and this inhibition may be due to the downregulations of curli genes (*csgA* and *csgB*) in EHEC (Kim et al., 2015) and biofilm-related adhesion genes (*icaA* and *sarA*) in *Staphylococcus* spp. (Jia et al., 2011). QS signaling controls EPS secretion, motility, protease production, and cell surface hydrophobicity (Li et al., 2014; Tan et al., 2014; Mizan et al., 2016; Pena et al., 2019), and in the present study, these virulence attributes of *A. tumefaciens* were remarkably and concentration-dependently inhibited by *t*-CNMA and its derivatives (Figures 4, 5). For *t*-CNMA, these effects have been suggested to be associated with the aldehyde group (Kot et al., 2015) as also shown for swimming motility of *E. coli* at 2.17 mM *t*-CNMA (Niu and Gilbert, 2004). Furthermore, it has been suggested that the presence of halogen (–Cl or –F) or nitro (–NO_2_) groups in *t*-CNMA increases its suppressive effects on bacterial virulence (Brackman et al., 2011; Nepali et al., 2018).

We also observed that 4-nitro CNMA, 4-chloro CNMA, and 4-fluoro CNMA reduced the lengths of *A. tumefaciens* cells (Figures 3G,I,K) as compared with non-treated (Figure 3C) and *t*-CNMA (Figure 3E) treated cells. However, we did not observe the t-CNMA-induced morphological distortions of *E. coli* and *S. aureus* cells reported by Shen et al. (2015). Furthermore, exposure to *t*-CNMA and the three derivatives had markedly impacted cell viability and growth (Figure 6), which could be associated with *t*-CNMA-induced reductions in intracellular pH (Oussalah et al., 2006), its interactions with membrane proteins (Mousavi et al., 2016), or its effects on cell membrane conductivity (He et al., 2019) or membrane lipid profiles (Wendakoon and Sakaguchi, 1995). In addition, the effects of –NO_2_, –Cl, and –F containing derivatives may have been influenced by the electronegativities of these groups (Shaikh et al., 2016; Doyle et al., 2019). *t*-CNMA has also been suggested to act as an ATPase inhibitor and inhibit enzymes involved in cytokine interactions (Shreaz et al., 2016). Furthermore, differences between the cellular uptakes of derivatives and their post-cellular uptake transformations may have modulated their effects.

**FIGURE 9:**
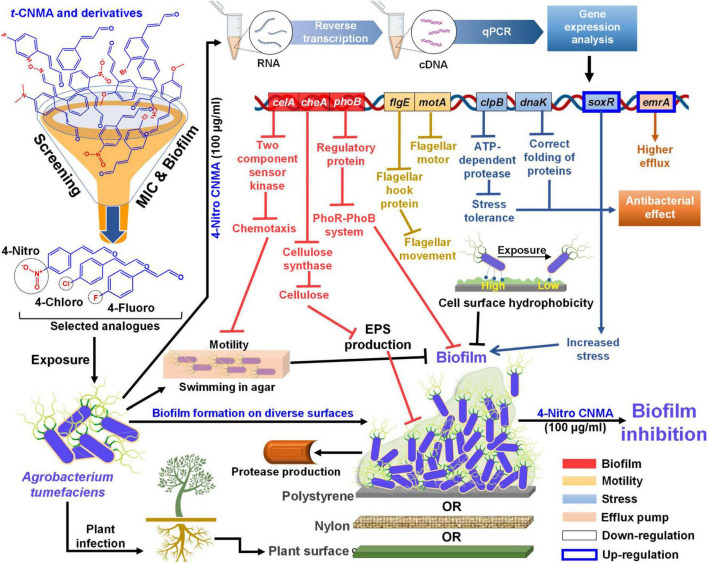
Mechanistic illustration of *A. tumefaciens* interaction with *t*-CNMA and its analogs specifically 4-nitro CNMA after screening based on MIC and biofilm inhibition percentages. *A. tumefaciens* was able to swim in agar by flagellar machinery, showed cell surface hydrophobicity, showed protease and EPS production, EPS and biofilm on three different surfaces nylon, polystyrene, and plant root (displayed beneath the biofilm). Selected derivatives inhibited these virulence factors and biofilm phenotype. Higher alterations in gene expressions were observed by 4-nitro CNMA than *t*-CNMA. Different color-coded gene boxes represent specific virulence phenotype (i.e. biofilm, motility, stress, and efflux), solid black bordered boxes show downregulation while blue bordered boxes show upregulation of genes. Arrows in different colors represent different group of genes and flat arrow ends stand for inhibition. cDNA, complimentary DNA; qPCR, quantitative polymerase chain reaction; MIC, minimum inhibitory concentration; EPS, exopolysaccharides.

No biofilm formation was observed on the root surfaces of *R. sativus* seedlings roots grown in the presence of 4-nitro CNMA, 4-chloro CNMA, or 4-fluoro CNMA (Figures 7G–O). A difference in biofilm volume on root surface and nylon surface was observed in non-treated groups where it was higher on nylon membranes possibly due to more firm attachment of cells on cellulose fibrils (Merritt et al., 2007; Heindl et al., 2014). The inhibition of *A. tumefaciens* biofilm formation on plant roots by CNMA derivatives has not been previously reported. However, biofilms of *Pseudomonas putida* KT2440 were reported to be dose-dependently inhibited by *t*-CNMA (Niu and Gilbert, 2004). *A. tumefaciens* utilizes adhesive pili, rhicadhesin, and chromosome-encoded factors in addition to universal forces like electrostatic and hydrophobic interactions and Van der Waals forces to attach to plant surfaces (Wheatley and Poole, 2018) and form biofilms. Our qRT-PCR data showed 4-nitro CNMA induced significant changes in the expressions of genes associated with motility (*flgE* and *motA*), biofilm formation (*celA*, *phoB*, and *cheA*), stress response (*clpB*, *dnaK*, and *soxR*), and efflux pump (*emrA*) (Figure 8) compared to *t*-CNMA. *A. tumefaciens* downregulated by 4-nitro CNMA induced a series of events that ultimately result in inhibition of bacterial adherence on surfaces, EPS, motility, biofilm as depicted in Figure 9. For example, *celA* encodes for a regulatory protein of two-component sensor kinase required for chemotaxis, therefore its downregulation reduces the chemotactic response of *A. tumefaciens* towards rhizospheric chemicals and prevent adherence to plant tissues (Figure 9). Another biofilm gene *cheA* encodes for cellulose synthase and induces EPS production, therefore its inhibition brings down the EPS matrix of biofilm. The increased transcription of *soxR* (stress related gene), sensing stress due to 4-nitro CNMA presence induced biofilm formation, however, other genes regulating the biofilm formation directly or indirectly were downregulated. The stress-related *clpB* and *dnaK* (or *Hsp70*) were downregulated and this ATP-dependent protease production and correct folding of proteins were compromised respectively that is suggestive of the possible antibacterial mechanism of 4-nitro CNMA.

Our findings suggest that the presence of strong electron-withdrawing groups at the fourth position of *t*-CNMA disrupts the biofilm formation mechanism of *A. tumefaciens*. The Ca^2+^ adhesion protein rhicadhesin has been proposed to play a role in *A. tumefaciens* attachment to plant surfaces (Swart et al., 1994), and it has been suggested that *t*-CNMA might similarly disrupt Ca^2+^ homeostasis in *Phytophthora capsici* (Hu L. et al., 2013). After evaluating the effects of *t*-CNMA, 4-nitro CNMA, 4-chloro CNMA, and 4-fluoro CNMA on *A. tumefaciens*, we suggest that structure-based activity factors and the presence of a conjugated aldehyde contribute to antibiofilm effects of *t*-CNMA. Xie et al. (2017) assigned the antifungal activity of *t*-CNMA and α-methyl CNMA to –CHO and –CH_3_ group at the ortho position of the aromatic ring. We found the presence of –NO_2_, –Cl, or –F at the para position had considerable effects on *A. tumefaciens* biofilms, and that 4-nitro CNMA had a greater suppressive effect on biofilm-associated genetic factors than *t*-CNMA (Figure 8).

## Conclusion

In summary, we evaluated the antibiofilm and antivirulence effects of *t*-CNMA and 4-nitro CNMA, 4-chloro CNMA, and 4-fluoro CNMA on *A. tumefaciens*. *t*-CNMA significantly reduced swimming motility, cell surface hydrophobicity, EPS secretion, and exo-protease production; however, these effects were considerably greater for 4-nitro CNMA and 4-chloro CNMA. We suggest the greater effects of these two derivatives on biofilm formation and growth may have been due to the presence of (i) a conjugated aldehyde group and (ii) an electron-withdrawing group like –NO_2_ at the para position. Also, qRT-PCR data showed 4-nitro CNMA downregulated the expressions of multiple biofilm formation associated genes, which shows CNMA derivatives target multiple processes and thus are unlikely to induce resistance in *A. tumefaciens*. Moreover, reductions in *A. tumefaciens* cell viability, growth, and root surface biofilm formation observed suggest that *t*-CNMA derivatives with –NO_2_, –Cl, or –F at position 4 on the aromatic ring provide an excellent starting point for the development of anti-*Agrobacterium* agents that effectively prevent crown gall disease.

## Data availability statement

The original contributions presented in this study are included in the article/Supplementary material, further inquiries can be directed to the corresponding authors.

## Author contributions

JL and J-HL: conceptualization, project administration, and funding acquisition. BA and AJ: methodology and software. BA, AJ, and JL: validation and writing the manuscript. JL: resources and supervision. All authors contributed to the article and approved the submitted version.
