# Supplementary material for: Inhibition of growth, biofilm formation, virulence, and surface attachment of Agrobacterium tumefaciens by cinnamaldehyde derivatives
Source: Front Microbiol. 2022 Oct 11;13:1001865. doi: 10.3389/fmicb.2022.1001865 (PMC9595724; doi:10.3389/fmicb.2022.1001865)
Supplement: Supplementary file 1 [file Data_Sheet_1.PDF]

# Supplementary Information

**Supplementary Table 1:** Biofilm, virulence, motility, efflux pump, and stress response related genes of *A. tumefaciens* with their primer sequences used for qRT-PCR.

| Gene | Function | Primer sequences |
|---|---|---|
| ***Motility*** | | |
| *flgE* | Flagellar hook protein | Forward 5′- TGG GAA GTA GCG GTT TAT CG -3′ |
| | | Reverse 5′- GGT CGA TAT TGA TCG CTT GC -3′ |
| *motA* | Flagellar motor protein | Forward 5′- GAA GCG CAC ATC GAT AAT CC -3′ |
| | | Reverse 5′- CGA TGA TGA TGA GAC GGA CA -3′ |
| ***Biofilm*** | | |
| *celA* | Cellulose synthase | Forward 5′- TGC CAC AAA CAT CGA AAA AG -3′ |
| | | Reverse 5′- ATT GTT CGG AAT TGG CAA AG -3′ |
| *cheA* | Chemotaxis protein | Forward 5′- GTG CCT TTG GTC TCG ATG AT -3′ |
| | | Reverse 5′- GCT TCC AGT TCC TTG ACG AG -3′ |
| *phoB* | Two component response regulator | Forward 5′- AGG TTC TGT CTT CGG TGC TG -3′ |
| | | Reverse 5′- TAG ATA TCG TGG CCC CAG AC -3′ |
| ***Virulence*** | | |
| *virE2* | ssDNA binding protein | Forward 5'- GGG ATG GAG TCG GCA ACT AC -3' |
| | | Reverse 5'- CAA TTG CTC CGG GAT ATC GA -3' |
| *chvE* | Virulence factor kinase | Forward 5′- GTT CTG CAG GCA ACT TCG AT -3′ |
| | | Reverse 5′- ACC TTG TCC ATT CCC ATC TG -3′ |
| *virE0* | Regulated protein | Forward 5′- TGC ACG GTG ATG ATG TTG ATC -3′ |
| | | Reverse 5′- ATC GAC GTA AGC GGC TTC AG -3′ |
| *virG* | Two component response regulator | Forward 5′- GTA GTC TGG CCA CGA AGT CC -3′ |
| | | Reverse 5′- ATG AGC GTC GAT CTT TGG TT -3′ |
| ***Stress response*** | | |
| *clpB* | Protease | Forward 5′- GCA AGC ATG TGG AGA AGG AT -3′ |
| | | Reverse 5′- CAG AAA CCG GTC GGT AAT GT -3′ |
| *dnaK* | Hsp70 chaperone protein | Forward 5′- GTC GAC CAA CGG TGA TAC C -3′ |
| | | Reverse 5′- GAT GAA CGG CAG GTT GAT TT -3′ |

| | | |
|---|---|---|
| *gsp* | Glutathionylspermidine synthase | Forward 5′- ATC TGC TCC CCA GCT TCT TT -3′<br>Reverse 5′- ATC CGA ACT CGC TCT TGA AC -3′ |
| *marR* | MarR family transcriptional regulator | Forward 5′- TCA GGC AAC GAC AAG TAC GA -3′<br>Reverse 5′- GTC TTC CCC AGC TCC CTT AC -3′ |
| *soxR* | SoxR family transcriptional regulator | Forward 5′- CAT CCA GCT CAG CAT CAT CA -3′<br>Reverse 5′- CAT ACG CGT CTG GAG CAT T -3′ |
| *hspAT2* | Small heat shock protein | Forward 5'-AAC CTT CTG ACC GTG ACT GG-3'<br>Reverse 5'-GCG ATT TTT CGT GGT CTC AT-3' |
| ***Efflux pump*** | | |
| *emrA* | Multidrug resistance efflux pump | Forward 5′- TCT CTT CTG CGA AAG CCA AT -3′<br>Reverse 5′- CGA GAT CAC CCG TCT GTA CC -3′ |
| *norM* | Multidrug efflux transporter | Forward 5′- CCG GAC AGT TCT TCT TCG TC -3′<br>Reverse 5′- CCC GAG ATA GAC CAG AAT GC -3′ |
| *ifeA* | Isoflavonoid-inducible efflux pump | Forward 5'-CAG CTG ACG ACA GCA CAG AT-3'<br>Reverse 5'-CAG CTG ACG ACA GCA CAG AT-3' |
| *ifeR* | Repressor controlling the expression of the putative *ifeABR* isoflavonoid efflux system | Forward 5'-GAT ATC GTT CTG GCG GAA AA-3'<br>Reverse 5'-GAT ATC GTT CTG GCG GAA AA-3' |
| ***Housekeeping*** | | |
| *16S rRNA* | 16S ribosomal RNA | Forward 5′- TGA CGA GTG GCG GAC GGG TG -3′<br>Reverse 5′- ATG CAG TTC CCA GGT TGA GC -3′ |

## Supplementary Figures

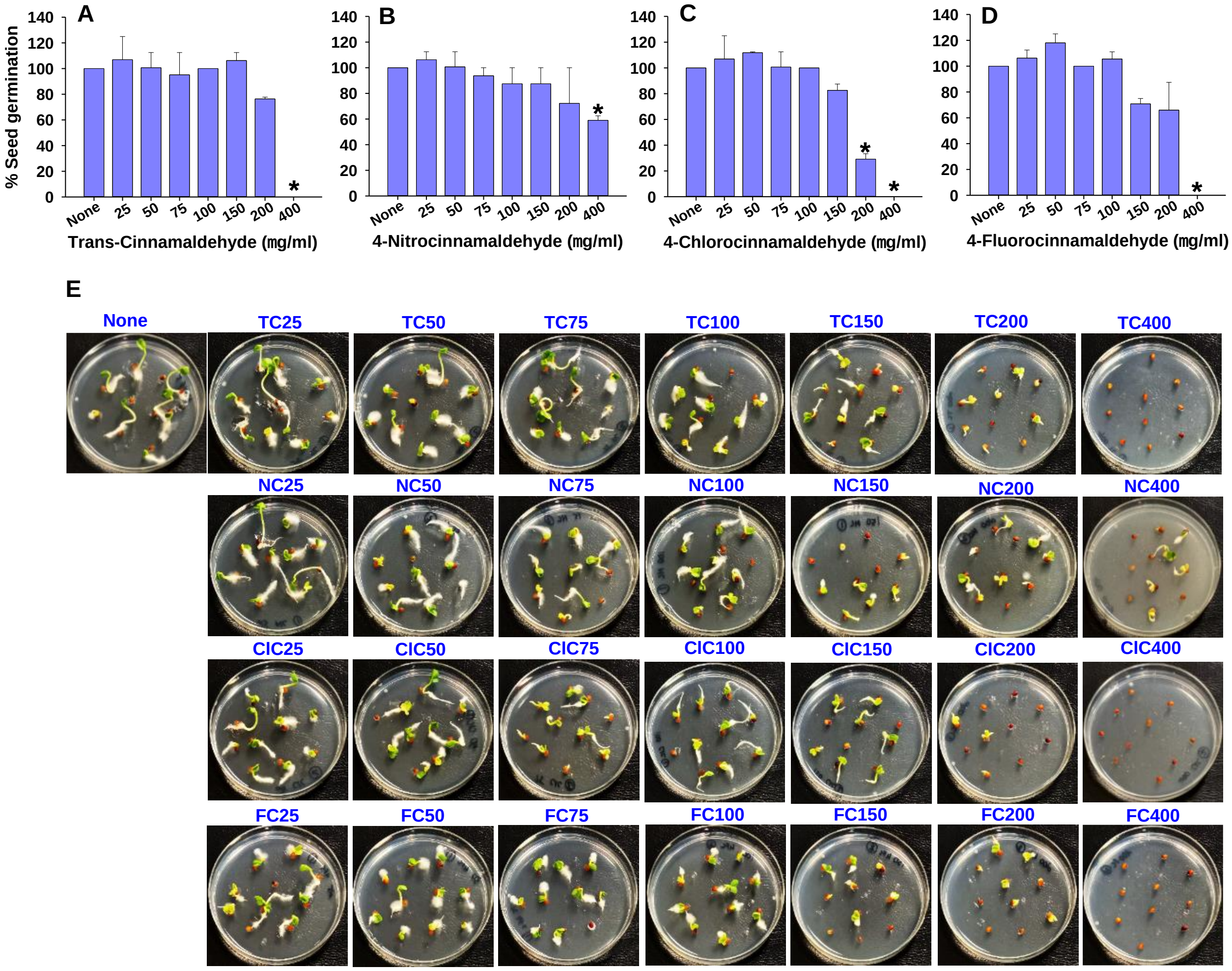


**Fig. S1** Effects of *t*-CNMA (A), 4-nitro CNMA (B), 4-chloro CNMA (C), and 4-fluoro CNMA (D) at 25-400 µg/ml on the germination of *R. sativus* seeds on semi-solid agar (0.7%) supplemented with MS medium (0.86 g/L) (E). Germination was recorded after incubation for four days at room temperature (≈25 °C). '*' denotes a significant difference as determined by the two-tailed t-test ($P \leq 0.05$).
